# Polyethersulfone Mats Functionalized with Porphyrin for Removal of Para-nitroaniline from Aqueous Solution

**DOI:** 10.3390/molecules24183344

**Published:** 2019-09-14

**Authors:** Chiara Maria Antonietta Gangemi, Mario Iudici, Luca Spitaleri, Rosalba Randazzo, Massimiliano Gaeta, Alessandro D’Urso, Antonino Gulino, Roberto Purrello, Maria Elena Fragalà

**Affiliations:** 1 Dipartimento di Scienze Chimiche, Università degli Studi di Catania, Viale A. Doria, 6-95100 Catania, Italy; gangemichiara@unict.it (C.M.A.G.); iudici.mario@virgilio.it (M.I.); lucaspitaleri@hotmail.it (L.S.); rrandazzo@unict.it (R.R.); gaetamassimiliano@libero.it (M.G.); agulino@dipchi.unict.it (A.G.); rpurrello@unict.it (R.P.); 2 INSTM UdR of Catania, Viale A. Doria, 6-95125 Catania, Italy

**Keywords:** porphyrin, para-nitroaniline, polyethersulfone, electrospinning, wastewater treatment

## Abstract

The dispersion of para-nitroaniline (p-NA) in water poses a threat to the environment and human health. Therefore, the development of functional adsorbents to remove this harmful compound is crucial to the implementation of wastewater purification strategies, and electrospun mats represent a versatile and cost-effective class of materials that are useful for this application. In the present study, we tested the ability of some polyethersulfone (PES) nanofibers containing adsorbed porphyrin molecules to remove p-NA from water. The functional mats in this study were obtained by two different approaches based on fiber impregnation or doping. In particular, meso-tetraphenyl porphyrin (H_2_TPP) or zinc(II) meso-tetraphenyl porphyrin (ZnTPP) were immobilized on the surface of PES fiber mats by dip-coating or added to the PES electrospun solution to obtain porphyrin-doped PES mats. The presence of porphyrins on the fiber surfaces was confirmed by UV–Vis spectroscopy, fluorescence measurements, and XPS analysis. p-NA removal from water solutions was spectrophotometrically detected and evaluated.

## 1. Introduction

Amines are used for many applications, but their release in the environment needs to be carefully monitored and controlled. Para-nitroaniline (p-NA) is a harmful compound due to its hematoxicity, splenotoxicity, hepatoxicity, and nephrotoxicity: accordingly, its dispersion in water strongly affects human health and represents a huge threat to the environment [1,2,3]. p-NA is resistant to chemical and biological oxidation degradation due to the presence of a nitro group linked to the aromatic ring. Therefore, the development of cost-effective membranes able to adsorb p-NA from aqueous solutions represents an alternative strategy to other treatments for wastewater purification (e.g., advanced oxidation processes, extraction, or biodegradation), and from this perspective, different types of adsorbents have been investigated and utilized to remove p-NA [4,5,6,7,8,9]. Recently, electrospun fibrous mats composed of a wide variety of polymers have been successfully applied in various fields, such as nanocatalysis [10,11,12], filtration [13,14,15,16], healthcare [17,18,19], biotechnology [20,21,22], security, and environmental control [23,24,25,26]. Polyethersulfone (PES) mats are largely used in membrane technology for water treatment due to their insolubility in water as well as their good mechanical and thermal properties [27,28,29]. Moreover, functionalized PES fibers have proved to be effective for applications in photocatalysis [24,30] and heavy metal removal [23,31,32,33], and electrospun fibers functionalized with porphyrins are suitable for the detection and removal of unwanted pollutants in air and water. In fact, porphyrins and metallo-porphyrins are widely used for developing sensing systems due to their coordination sites for axial ligation and significant optical modifications which provide evidence of successful core complexation [34]. In particular, zinc(II) meso-tetraphenyl porphyrin (ZnTPP) and meso-tetraphenyl porphyrin (H_2_TPP) have been used to optically detect NH_3_ and gaseous amines [35,36]. Therefore, the immobilization of these versatile macrocycles on surfaces is fundamental to obtain active layers devoted to molecular recognition in gas or liquid phase, and various techniques have been proposed to obtain porphyrin layers on substrates [37,38].

**Figure 1:**
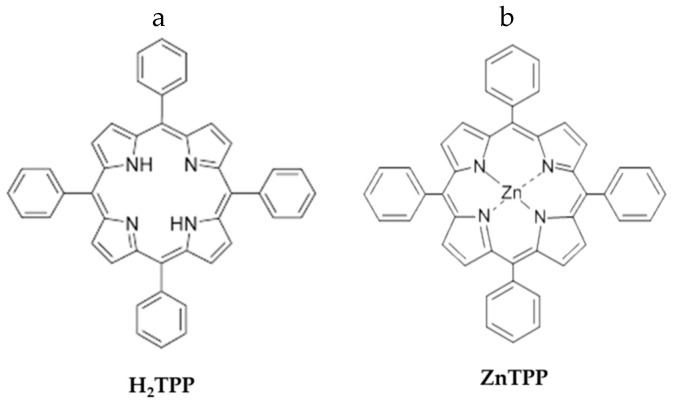
(**a**) Structure of meso-tetraphenyl porphyrin; (**b**) Structure of zinc(II) meso-tetraphenyl porphyrin.

In this general panorama, the aim of our work was to test the ability of porphyrin-modified PES nanofibers to remove p-NA from water. To our knowledge, little data reporting the use of electrospun mats as adsorbents to remove p-NA from water is available. In particular, herein we functionalized PES fibers with meso-tetraphenyl porphyrin (H_2_TPP) or zinc(II) meso-tetraphenyl porphyrin (ZnTPP) to exploit the ability of these porphyrins to coordinate amino and/or nitro groups of p-NA (Figure 1).

Porphyrin-loaded fibers were prepared using two different approaches: H_2_TPP or ZnTPP were immobilized on the surface of PES mats by i) dip-coating in porphyrin solutions (impregnation); or ii) by adding H_2_TPP or ZnTPP to the PES electrospinning solution to obtain porphyrin-doped PES mats. The presence of porphyrins on the fiber was confirmed by UV–Vis spectroscopy, fluorescence measurements, and XPS analysis, and p-NA removal from water solutions was spectrophotometrically detected and evaluated.

## 2. Results and Discussion

**Figure 2:**
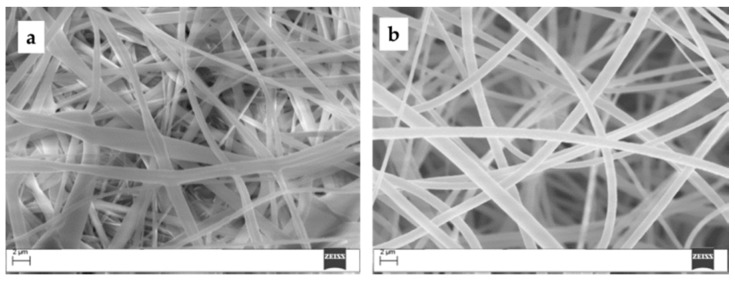
SEM images of polyethersulfone (PES) mats (**a**) before and (**b**) after dipping in a 5 μM H_2_TPP solution in toluene.

Fiber impregnation was achieved by dip-coating PES electrospun mats in 5 μM H_2_TPP or ZnTPP toluene solutions. In this case, porphyrin adsorption was promoted by non-covalent interactions, which depend on several factors, including the specific surface area and mobility of dye molecules both in the liquid phase and in the interior of the polymeric scaffold. ZnTPP and H_2_TPP are readily soluble in toluene whilst PES mats are only moderately soluble. SEM analysis (Figure 2) demonstrated how the mats’ integrity was maintained after 2 h dipping in toluene-porphyrin solutions.

Moreover, impregnated mats were also extensively washed and sonicated in water after dip-coating, and UV–Vis measurements of the washing solution ruled out any porphyrin release in water, thus confirming the robustness of this non-covalent grafting.

**Figure 3:**
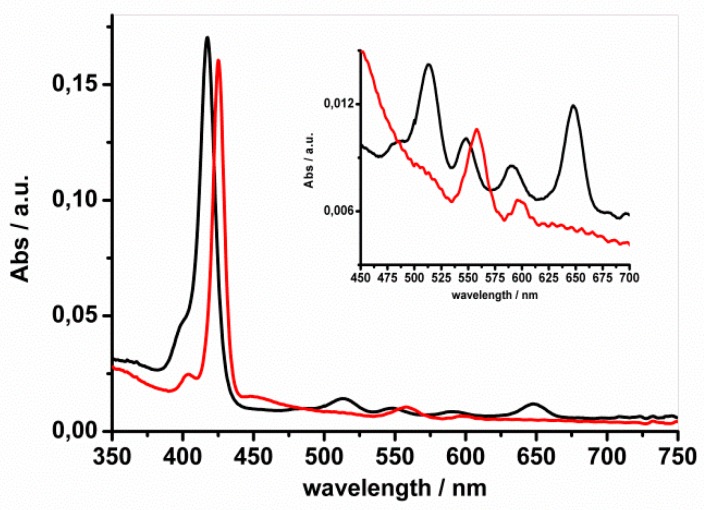
UV–Vis spectra of toluene:DMF (50:50 *v*:*v*) solutions in which PES mats impregnated with H_2_TPP (black line) or ZnTPP (red line) were dissolved. The inset shows the Q-bands region in detail.

To control the amount of adsorbed porphyrin, the fibers were dissolved in a toluene:DMF (50:50 *v*:*v*) solution and the UV–Vis spectrum (Figure 3) for H_2_TPP showed an intense Soret band at 417 nm and four Q-bands at 513, 548, 590, and 647 nm, while for ZnTPP the Soret band was at 425 nm and the two expected Q-bands were at 558 and 598 nm [39].

The porphyrin concentration on mats (having 2 × 2 cm^2^ surface area and 2.4 mg weight, dissolved in 2.5 mL of toluene:DMF 50:50 *v*:*v*) was spectrophotometrically estimated to be 0.32 μM and 0.30 μM for H_2_TPP and ZnTPP, respectively (~0.02% *w*/*w* for both H_2_TPP and ZnTPP).

**Figure 4:**
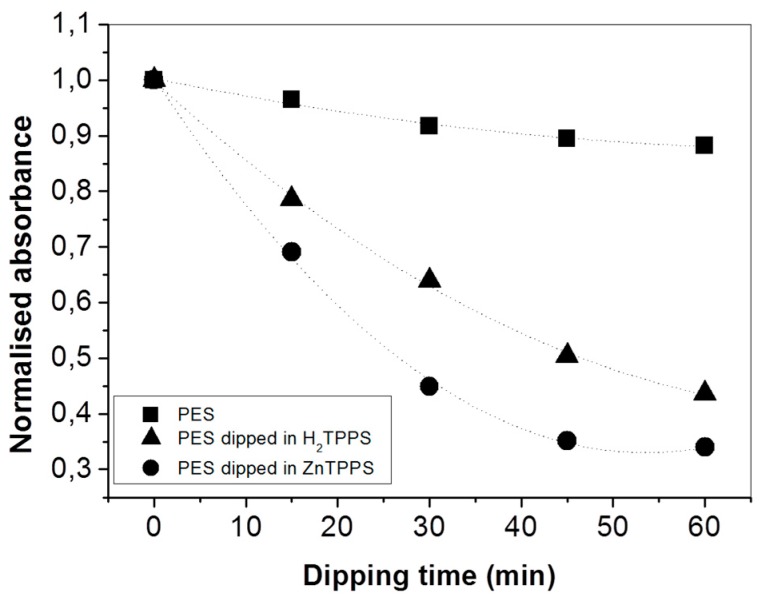
Normalized absorbance variation at λ = 381 nm of a 5 µM para-nitroaniline (p-NA) water solution upon increasing the dipping time of untreated (black squares) and H_2_TPP- (black triangles) and ZnTPP-treated (black circles) PES mats.

The presence of p-NA in water can be spectrophotometrically estimated using the band at 381 nm (Figure S1) and, accordingly, the ability of porphyrin-impregnated PES mats to interact with p-NA can be spectrophotometrically studied by measuring the absorbance decrease at λ = 381 nm after the dipping of untreated and porphyrin-treated PES mats (Figure S2). Figure 4 shows the trend of normalized absorbance upon increasing the mats’ dipping time in the p-NA solution: untreated PES fibers showed negligible p-NA removal (less than 10% after 2 h), whilst a signal reduction up to 60% in the presence of H_2_TPP PES mats and 70% for ZnTPP PES mats was observed (corresponding to an average p-NA residual concentration of p-NA in solution of about 2 μM).

To explain this behavior, we can invoke the ability of H_2_TPP and ZnTPP to coordinate nitro and amino groups [40,41]. However, it is important to note that the nitro group reduces the NH_2_ basicity in p-NA and, accordingly, a preferential coordination of Zn^2+^ with the –NO_2_ oxygen atoms, rather than with –NH_2_ nitrogen, can be hypothesized. Note that the use of polar solvent has an important effect on the Lewis acid–base interaction between Zn^2+^ and the donor atoms, and the presence of water can contribute to enhance π−π interactions between aromatic groups [42]. The presence of p-NA on the fiber surface was further supported by XPS analyses, which indicated an approximate increase of 65% in the nitrogen concentration upon p-NA (data not showed).

**Figure 5:**
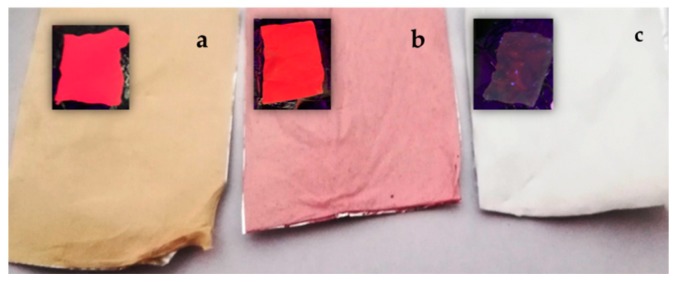
Pictures of (**a**) electrospun ZnTPP/PES, (**b**) electrospun H_2_TPP/PES, and (**c**) impregnated H_2_TPP/PES mats. Images under UV lamp irradiation are shown in the inset.

The reported data demonstrate the importance of H_2_TPP and ZnTPP in promoting p-NA adsorption on PES fibers, but the dip-coating procedure remains time-expensive and suffers from a lack of reproducibility in terms of the homogeneity of distribution of porphyrin on PES surfaces, as demonstrated by the fluorescence response (Figure 5).

The second strategy we used to fabricate hybrid porphyrin/PES mats was based on the electrospinning of porphyrin and PES solutions, thus leading to porphyrin-doped mats. This approach guarantees a better control of the amount of porphyrin in the PES matrix as well as a high fabrication yield (a 20 × 40 cm^2^ foil can be prepared in less than 1 h). Porphyrin/PES electrospun mats and impregnated fibers could be easily distinguished by their different coloration (Figure 5a–c), and were all characterized by strong fluorescence emissions under UV irradiation (see inset).

The amount of porphyrin in electrospun prepared mats (Figure 5a,b) higher than that obtained for impregnated mats (Figure 5c) was responsible for the different coloration of the fibers.

The photoluminescence spectra (*λ_ex_* = 422 nm) of electrospun H_2_TPP/PES or ZnTPP/PES dissolved in toluene:DMF (50:50 *v*:*v*) solutions showed two emissions at 650 and 715 nm for H_2_TPP/PES mats and at 600 and 650 nm for ZnTPP/PES mats (Figure S3).

**Figure 6:**
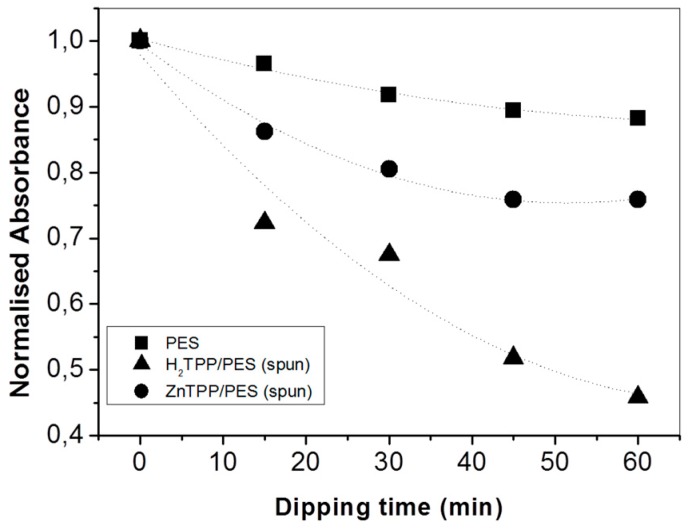
Normalized absorbance decrease of p-NA solution (λ = 381 nm) upon increasing the dipping of the time of untreated and porphyrin/PES electrospun mats. The initial concentration of p-NA was 5 µM. Squares, circles, and triangles refer to untreated PES, ZnTPP/PES, and H_2_TPP/PES fibers, respectively.

UV–Vis analysis of an aqueous 5 µM p-NA solution after fiber immersion (Figure 6) indicated that p-NA preferentially absorbed on H_2_TPP/PES fibers.

Note that ZnTPP usually adopts a five-coordinate structure by the axial complexation of a solvent molecule containing electron donors such as N, O, and S [43,44]. Accordingly, it is reasonable to assume changes in the electrospun solution properties (i.e., surface tension, viscosity, conductivity) arising from different interactions of H_2_TPP or ZnTPP with the DMF solvent and PES sulfonic groups, and these effects might be responsible for the different geometrical arrangement and surface availability of porphyrins on the polymeric matrix. As a result, the Zn^2+^ in the electrospun fiber is probably less available to axially coordinate with p-NA.

**Figure 7:**
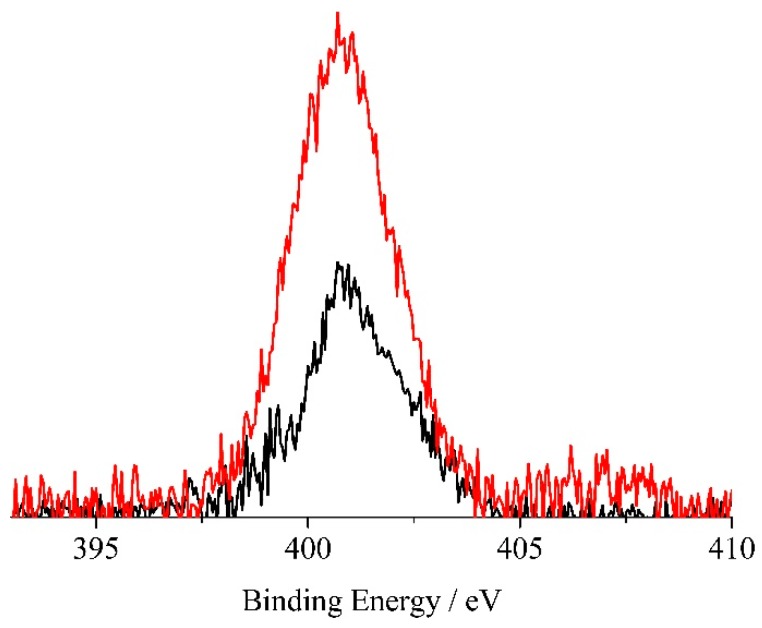
Al Kα excited XPS of a representative PES electrospun mat with H_2_TPP before (black line) and after sensing of the p-NA (red line) in the N 1s binding energy region. The signal intensities have been normalized to results of the atomic concentration analysis.

The XPS analysis performed on electrospun H_2_TPP/PES and ZnTPP/PES confirmed the presence of p-NA on the fiber surfaces. In particular, Figure 7 shows the XPS spectrum of the H_2_TPP/PES before and after sensing of the p-NA, in the N 1s binding energy region. As already reported in a large number of studies, XPS ionizations of the four pyrrole nitrogen atoms in porphyrins, for symmetry restrictions, show two N 1s signals having a 1:3 intensity ratio [45,46,47,48,49,50,51]. This behavior is well documented by literature data, and finds a counterpart in the present spectrum consisting of the convolution of two components at 399.3 and 400.8 eV, with a 1:3 intensity ratio [45]. After p-NA sensing, this spectrum showed major changes, mainly due to the intensity increase of the low binding energy component now at about 399.6 eV. Moreover, there is also evidence of a very small feature at about 407 eV, in tune with the presence of the –NO_2_ group of the p-nitroaniline [45]. In this context, it has already been reported that –NO_2_ groups reduce to –NH_2_ during XPS measurements because of the X-ray irradiation, and this observation is in strong agreement with both the observed intensity increase of the low binding energy component at about 399.6 eV, and with the very low intensity of the feature at 407 eV [52]. In addition, we noted a 100% atomic concentration increase of the N 1s signal, confirming the increase of nitrogen-containing species on the PES mats upon interaction with the p-NA.

**Figure 8:**
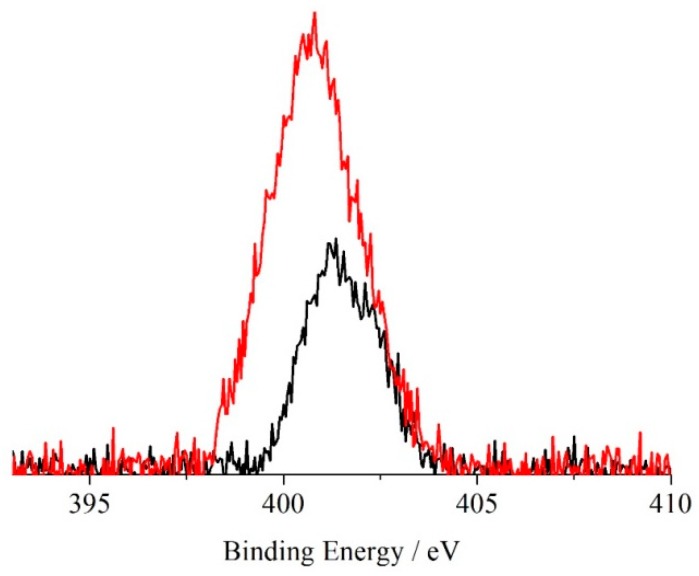
Al Kα excited XPS of a representative PES electrospun mat with ZnTPP before (black line) and after sensing of the p-NA (red line) in the N 1s binding energy region. The signal intensities have been normalized to results of the atomic concentration analysis.

Figure 8 shows the XPS spectra of the ZnTPP/PES mats before and after sensing of the p-NA in the N 1s binding energy region. The increased symmetry of this porphyrin (group D_4h_) is evident in the XPS of the N 1s states that now show a unique band at 401.1 eV [45]. Upon contact with the p-NA, we noted a lower binding energy shift of the whole spectrum that now peaked at 400.6 eV [52]. Again, we also noted a 100% atomic concentration increase of the overall N 1s signal. These results strongly confirm that the present porphyrin-doped PES mats are well-suited to sense p-NA.

## 3. Materials and Methods

Polyethersulfone was purchased from Sigma Aldrich and used without purification. 5,10,15,20-tetrakisphenyl-21H,23H-porphyrin (H_2_TPP; MW = 614.74 g/mol) and 5,10,15,20-tetrakisphenyl-zinc (II) (ZnTPP; MW = 678.1 g/mol) porphyrin were purchased from Sigma Aldrich. Anhydrous *N*,*N*-dimethylformamide (DMF) and toluene were purchased from Sigma Aldrich and used as received.

### 3.1. Porphyrin Impregnation of PES Mats by Dip-Coating

The H_2_TPP extinction (ε) coefficient at 418 nm (in toluene) is 525,000 M^−1^cm^−1^, while for ZnTPP (in toluene) at 422 nm ε is 537,000 M^−1^cm^−1^ [29]. Samples of PES fibers having a 2 × 2 cm^2^ dimension were dipped for times ranging from 30 min up to 2 h in 10 mL of 5 μM H_2_TPP or ZnTPP toluene solutions. No stirring was applied. The absorbance of these porphyrin solutions was checked every 15 min. After dipping, mats were washed in water and dried in air. There was no release of porphyrins in water, even after 1 h sonication, as confirmed by UV–Vis spectra.

### 3.2. Porphyrin/PES Electrospinning

Briefly, 2.5 g of PES and appropriate aliquots of ZnTPP or H_2_TPP, corresponding to 6% *w*/*w*, were dissolved in 5 mL of toluene and 5 mL of DMF, respectively, under vigorous stirring at 40 °C to obtain the electrospinning solutions. Each spinning solution was placed in a syringe and pumped with a rate of 30 μL/min. A high-voltage power supply was used to generate a potential difference of 24 kV between the spinneret and an aluminum-foil-covered grounded metallic drum, rotating at 200 rpm, placed at a working distance (WD) of 15 cm. All the experiments were carried out at 25° C. After electrospinning, fibers were dried in air for 24 h.

### 3.3. Para-Nitroaniline (p-NA) Absorption Measurements

Dipping solutions of p-NA [5 μM] were prepared in ultrapure water and checked by UV–Vis absorption spectra (ε = 13,500 M^−1^cm^−1^). The absorption measurements were performed by dipping porphyrin functionalized mats (electrospun or impregnated) of dimensions 2 × 2 cm^2^ into 10 mL of this aqueous p-NA solution (pH = 7). Absorption spectra of treated p-NA solutions were recorded every 15 min after mats’ dipping.

### 3.4. Measurements

Nanofiber morphologies and diameters were evaluated using a field emission scanning electron microscope (Zeiss Supra 55-VP FEGSEM). To spectroscopically evaluate the presence of porphyrins on impregnated or electrospun mats, samples were dissolved in 1:1 toluene:DMF solutions. Absorbance intensities of the 3.6 μM solutions of ZnTPP in the presence and absence of PES were compared (Figure S4) to determine the role of PES in the porphyrin absorbance. A Jasco V-560 UV–Vis spectrophotometer equipped with a 1 cm path-length cell was used for the UV–Vis measurements. For fluorescence measurements a Fluorolog FL-11 Jobin-Yvon Horiba was used. Each experiment was carried out at 25 °C. X-ray photoelectron spectra (XPS) were measured with a PHI 5600 Multi Technique System (base pressure of the main chamber 3 × 10^−8^ Pa) [45,53]. Samples were excited with Al Kα X-ray radiation using a pass energy of 5.85 eV. The instrumental energy resolution was ≤0.5 eV. XPS peak intensities were obtained after a Shirley background removal [45,53]. Spectral calibration was achieved by fixing the main C 1s peak at 285.0 eV [45,53]. Structures due to the Al-Kα X-ray satellites were subtracted prior to data processing. The atomic concentration analysis was performed by taking into account the relevant atomic sensitivity factors. The samples were repetitively sonicated/washed in water before XPS analyses.

## 4. Conclusions

The combination of H_2_TPP or ZnTPP with PES electrospun mats resulted in the effective removal of p-NA from water. Both adopted fabrication approaches confirmed the role of porphyrins in p-NA immobilization on PES surfaces with respect to bare PES mats, but further investigations are required to improve the adsorption capacity in order to have a reliable comparison with other adsorbents [4,5,6,7,8,9,54]. XPS analysis confirmed the presence of p-NA on the fiber surfaces: due to the sampling depth of this technique (limited to a few nanometers), no significant differences between the surface compositions of H_2_TPP/PES and ZnTPP/PES mats were detected. Nevertheless, UV–Vis analysis pointed out the role of porphyrin organization on the removal efficiency. In particular, ZnTPP-impregnated mats showed better performances than their H_2_TPP-treated counterparts. In contrast, H_2_TPP/PES electrospun mats were remarkably more efficient than ZnTPP/PES mats. These results indicate a limited availability of Zn^2+^ ions for the axial coordination of N donor atoms in electrospun mats. Note that the p-NA absorption efficiency of both impregnated and electrospun H_2_TPP containing mats was comparable, despite the different amounts of incorporated porphyrins. Accordingly, we suppose that the interaction of p-NA with H_2_TPP depends only on the amount of porphyrin located on the surface, which should be similar in both types of fiber. The study of the spatial organization of porphyrin during electrospinning, and on the influence of the working parameters on mats’ response to the detection and removal of p-NA, is an ongoing work.

## Supplementary Materials

The following are available online at https://www.mdpi.com/1420-3049/24/18/3344/s1, Figure S1: UV–Vis spectrum of p-NA in water (5 μM) before porphyrin-treated mat dipping. Figure S2: UV–Vis spectrum of p-NA in water (5 μM) before (black line) and after dipping (1 h) with porphyrin-treated mats H_2_TPP/PES (green line) or with ZnTPP/PES (red line). Figure S3: Absorption and emission spectra (λ_ex_ = 422 nm) of electrospun H_2_TPP/PES and ZnTPP/PES dissolved in toluene:DMF. Figure S4: UV spectra of 1.5 μM ZnTPP toluene:DMF solution in absence and presence of PES.
