# Supplementary material for: Polyethersulfone Mats Functionalized with Porphyrin for Removal of Para-nitroaniline from Aqueous Solution
Source: Molecules. 2019 Sep 14;24(18):3344. doi: 10.3390/molecules24183344 (PMC6766941; doi:10.3390/molecules24183344)
Supplement: Supplementary file 1 [file molecules-24-03344-s001.pdf]

Supplementary Materials

Polyethersulfone mats functionalized with porphyrin for removal of p-NA from aqueous solution.

Chiara Maria Antonietta Gangemi,[1] Mario Iudici,[1] Luca Spitaleri,[1] Rosalba Randazzo,[1] Massimiliano Gaeta,[1] Alessandro D'Urso,[1] Antonino Gulino, [1,2] Roberto Purrello[1] and Maria Elena Fragalà [1,2*]

[1] Dipartimento di Scienze Chimiche, Università degli Studi di Catania, Viale A. Doria, 6 - 95100 Catania (Italy)

[2] INSTM UdR of Catania,Viale A. Doria, 6 - 95125 Catania (Italy)

* Correspondence: me.fragala@unict.it; Tel.: +390957385149

Received: date; Accepted: date; Published: date

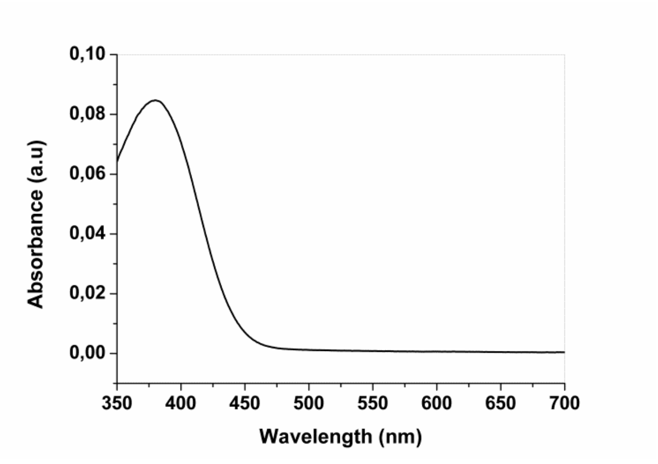

Figure S1. UV-Vis spectrum of p-NA in water (5 μM) before porphyrin treated mats dipping.

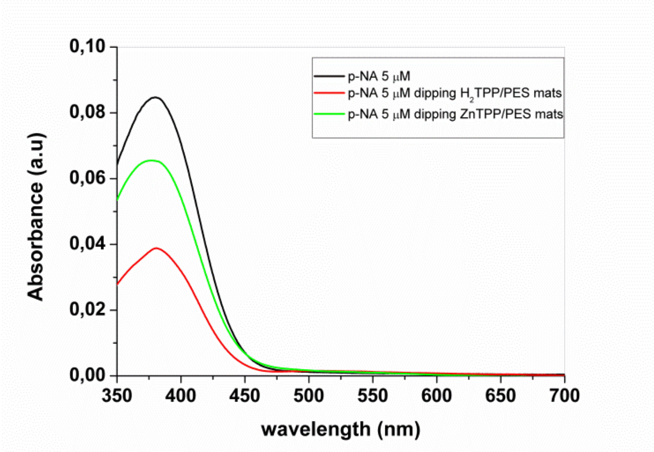

Figure S2. UV-Vis spectrum of p-NA in water (5 μM) before (black line) and after dipping (1 h) with porphyrin treated mats H_2TPP/PES (green line) or with ZnTPP/PES (red line).

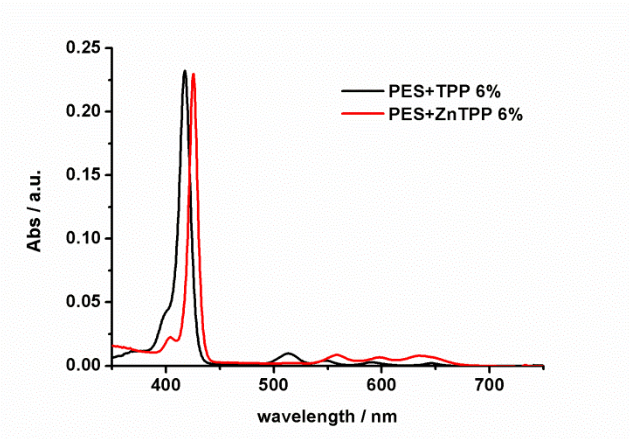

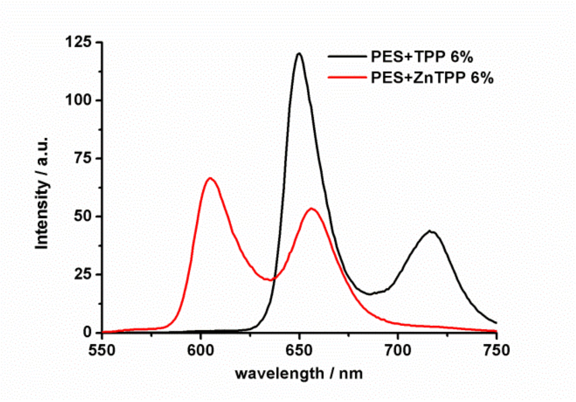

Figure S3. Absorption and emission spectra (λ_{ex} = 422 nm) of electrospun H_2TPP/PES and ZnTPP/PES dissolved in toluene:DMF.

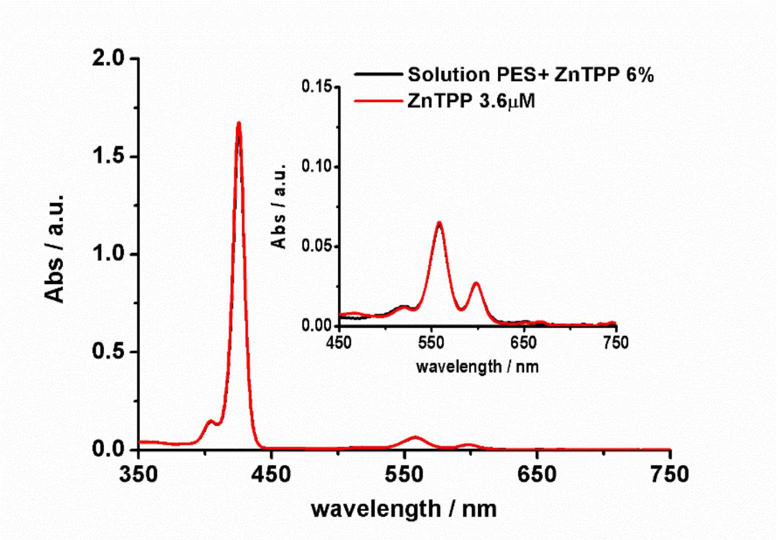

Figure S4. Absorption spectra of ZnTPP 3.6 μM in toluene:DMF (red curve) and in the presence of PES (black curve).
